# MRC-DETR: A High-Precision Detection Model for Electrical Equipment Protection in Power Operations

**DOI:** 10.3390/s25134152

**Published:** 2025-07-03

**Authors:** Shenwang Li, Yuyang Zhou, Minjie Wang, Li Liu, Thomas Wu

**Affiliations:** School of Electrical Engineering, Guangxi University, Nanning 530004, China; lishenwang@gxu.edu.cn (S.L.); 13906329750@163.com (Y.Z.); w13788112519@163.com (M.W.); liliu.ee@gxu.edu.cn (L.L.)

**Keywords:** deep learning, Power Engineering Personal Protective Equipment (PEPPE), DETR, real-time object detection, model deployment

## Abstract

Ensuring that electrical workers use personal protective equipment (PPE) correctly is critical to electrical safety, but existing detection methods face significant limitations when applied in the electrical industry. This paper introduces MRC-DETR (Multi-Scale Re-calibration Detection Transformer), a novel framework for detecting Power Engineering Personal Protective Equipment (PEPPE) in complex electrical operating environments. Our method introduces two technical innovations: a Multi-Scale Enhanced Boundary Attention (MEBA) module, which significantly improves the detection of small and occluded targets through optimized feature representation, and a knowledge distillation strategy that enables efficient deployment on edge devices. We further contribute a dedicated PEPPE dataset to address the lack of domain-specific training data. Experimental results demonstrate superior performance compared to existing methods, particularly in challenging power industry scenarios.

## 1. Introduction

The use of personal protective equipment (PPE) by electrical workers plays a crucial role in ensuring the safety of workers during electrical operations and maintaining the normal operation of the power grid. The work environment for live-line workers is highly hazardous. According to a report by the U.S. Occupational Safety and Health Administration (OSHA), there were 1254 live-line work accidents involving 1399 workers in the United States between 2010 and 2022. Among these, 817 were injured, and 582 workers died (a fatality rate of 41.6%) [1]. This not only poses a threat to workers’ lives but also causes immeasurable losses to the power supply system. However, even after relevant departments provide safety training to live-line workers, violations may still occur due to subjective and objective factors [2,3]. Therefore, an external supervision method is needed to confirm whether personnel are properly wearing protective equipment, thereby reducing the safety hazards associated with live-line work.

Currently, personnel equipment detection primarily relies on sensor-based and image-based methods. Sensor-based detection is divided into traditional PPE detection and intelligent PPE detection. Traditional PPE detection includes scanning radio frequency identification (RFID) tags attached to personal protective equipment using RFID technology [4,5,6,7] and detecting the wearing status of equipment by workers using pressure [8] or human body sensors [9]. Intelligent PPE detection can monitor physiological parameters, environmental conditions, and potential hazards, providing workers with real-time feedback and early warning systems [10]. Examples include an intelligent platform for workplace safety using the Jetson-based monitoring of environmental variables in the workplace [11], a safety monitoring system using a Raspberry Pi (Sony UK TEC, Pencoed, UK) to monitor individuals’ physiological parameters and environmental conditions [12], and the construction of a dataset of skeletal information to determine risky behaviors using the ST-GCN algorithm [13]. However, the actual deployment effectiveness of these systems in power work environments remains to be verified, especially given the limited signal transmission range during high-altitude operations and the susceptibility of communication devices to interference in high-electric-field environments. Additionally, sensor-based methods incur high costs in terms of installation and maintenance [14] and have low detection efficiency (a single set of equipment can only be applied to a single worker, requiring multiple sets for multi-person operations).

In contrast, visual-based methods use cameras to record images or videos of the work site, which are then analyzed to verify PPE compliance. This approach provides richer information about the scene, enabling a faster, more accurate, and comprehensive understanding of complex construction sites [15]. Traditional visual method examples include, but are not limited to, video-based monitoring methods that use facial features, motion, and color information [16], image-based edge detection [17], and directional gradient histogram (HOG) methods [18] to detect safety helmets. Other examples use HOG-based features and machine learning algorithms, such as support vector machines (SVM) [19] and k-nearest neighbors (kNN), to detect whether a person is wearing a safety vest [20].

Recent advances in deep learning have facilitated the application of convolutional neural networks (CNNs) and object-detection models for PPE detection. Existing studies contain approaches based on improved target detection algorithms, such as Faster R-CNN and different variants of the YOLO family (including YOLOv5-CBAM-DCN, YOLOX, and optimized YOLOv7, etc.) in [21,22,23,24,25]; and, secondly, detection systems incorporating post-processing show enhanced utility such as the fusion of pose detection to recognize offending movements, etc. [26]; in addition, some innovative approaches such as multi-task learning frameworks and graph neural networks provide new solutions for small-sample scenarios and specific protective equipment detection [27,28]. These studies not only promote the development of protective-equipment-detection technology but also provide important references for model selection and optimization in practical applications [14,29].

**Figure 1:**
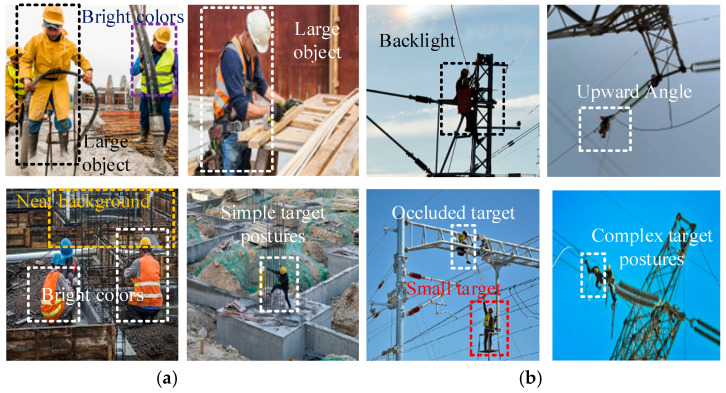
(**a**) PPE image features, (**b**) PEPPE image features.

However, a major limitation of existing research is the insufficient attention paid to the detection of personal protective equipment (PPE) in the power industry. This sector typically involves outdoor working conditions, high altitudes, and exposure to environmental complexity and equipment variability. Most models are developed and validated in relatively controlled environments or using generic PPE datasets, which limits their applicability in power engineering environments. As shown in Figure 1, PPE detection in power operation scenarios differs significantly from that in general construction environments. Images are typically taken from overhead or downward angles, with workers being small targets with complex postures and backgrounds often containing elements such as power towers and cables. This makes traditional PPE-detection models unsuitable for PPE identification in the power industry.

There also exist PPE-detection methods applicable to the power industry (including scenarios such as work-at-height and power operations), such as PPE-detection models for work-at-height based on the UAV perspective [30,31] and PPE-detection based on high-voltage scenarios [32]. Other similar studies such as the detection models of safety equipment applied to construction sites working at a height [33,34] and a variety of high-risk scenarios, such as high-altitude work zones, power chemical, and tunnel underground work [35], also provide research ideas for PPE detection in the power industry. These methods perform well in the scenarios they address but are limited by the number of targets they focus on (according to the China Electric Power Construction Safety Work Procedures, high-altitude workers must wear safety helmets, insulated clothing, etc., in addition to safety belts; according to the US NFPA 70E (US Electrical Safety Standard) [36], rubber insulated gloves and sleeves, insulated boots, hard hats, safety goggles/masks, etc., are explicitly required in arc hazard zones. Therefore, the aforementioned object-detection systems are not well-suited for practical applications) are relatively limited [30,31,32,33,34], their effectiveness in other high-altitude work environments (such as power tower maintenance or bridge construction) has not been thoroughly validated [35], and no practical method has been proposed to effectively address these applications. Therefore, it is necessary to develop a PPE detection model specifically tailored to power operation scenarios, which should have the following characteristics: 1. applicable to power operation scenarios, 2. capable of detecting multiple types of safety protective equipment, 3. capable of meeting real-time detection requirements.

**Figure 2:**
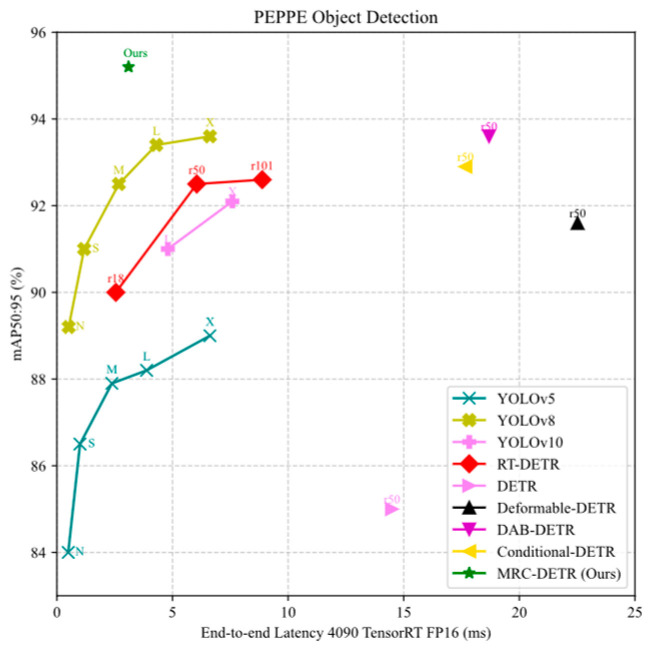
Comparison of different models in mAP50:95 and latency in PEPPE datasets.

In this work, we introduce the concept of Power Engineering Personal Protective Equipment (PEPPE) detection and propose a novel model—Multi-scale Re-Calibration Detection Transformer (MRC-DETR)—tailored to the operational characteristics and safety requirements of power environments. Building on the DETR architecture, MRC-DETR incorporates structural enhancements to improve its ability to detect small objects, handle occlusions, and identify various PEPPE items under dynamic and high-risk conditions. Our model achieved good accuracy and detection performance based on the PEPPE dataset, as shown in Figure 2.

The main contributions of this work can be summarized as follows:

1. We designed a Multiscale Enhanced Boundary Attention (MEBA) module that optimizes the model’s feature representation by integrating multi-scale features, significantly improving the model’s ability to represent small and occluded targets in complex scenes.

2. We proposed the Multi-scale Re-Calibration Detection Transformer (MRC-DETR), which uses knowledge distillation methods to improve the performance of lightweight models for real-time detection in resource-constrained environments.

3. We established a PEPPE dataset focused on personal protective equipment for electrical engineering, which includes multiple types of personal protective equipment for electrical engineering. Finally, we deployed the optimized model on edge devices to achieve real-time PEPPE detection in electrical work scenarios, laying the technical foundation for future practical applications.

## 2. Related Works

### 2.1. Object Detection Based on a Transformer

Object detection is a core task in computer vision, aimed at locating all objects in an image and identifying their categories [37]. Currently, mainstream object-detection methods are primarily based on two types of architectures: convolutional neural networks (CNNs) and transformers.

Over the past decade, CNN architectures have dominated the research landscape in object detection, evolving into two primary paradigms: two-stage and one-stage detectors. Two-stage methods such as the R-CNN [38], Fast R-CNN [39], and Faster R-CNN [40] series achieve detection through two stages: candidate region extraction and classification regression. While these methods offer high accuracy, they are limited in inference speed [41]. In contrast, one-stage detectors like YOLO (including the YOLO series of object detection models [42,43,44,45] and SSD [46]) perform end-to-end bounding box regression and classification directly on the image, significantly improving the detection speed while achieving an acceptable trade-off in accuracy [47]. However, CNN-based methods still have limitations in modeling long-range dependencies, global context awareness, and object relationship representation, which restrict their performance in complex scenarios.

To address these issues, the transformer architecture was introduced into object detection tasks, giving rise to the DETR (DEtection TRansformer) [48] method. It abandons traditional anchor-based designs and post-processing steps (such as NMS), directly predicting object bounding boxes and categories through an encoder–decoder structure. DETR’s advantages include strong global modeling capabilities, a simple structure, and high end-to-end consistency during training [49]. However, DETR’s training process is relatively slow, converges at a slower rate than CNN detectors, and performs poorly in detecting small objects.

To address these shortcomings, researchers have proposed various DETR variants. For example, Deformable DETR [50] introduces a deformable attention mechanism to focus on local regions, significantly accelerating the convergence speed; DN-DETR [51], DINO [52], and others improve matching mechanisms and decoding strategies, further enhancing the detection accuracy and training efficiency [53]; RT-DETR [54] maintains the end-to-end detection structure while significantly improving inference efficiency through an efficient architectural design.

Overall, object-detection technology is gradually transitioning from CNN architectures, which are efficient but have limited local modeling capabilities, to transformer architectures that combine global perception with end-to-end training advantages. The combination and complementarity of the two has also become an important research direction in recent years [55].

### 2.2. Knowledge Distill

Knowledge Distillation (KD) is a model compression and transfer learning technique for which the core idea is to guide the learning of a student model through a teacher model, thereby significantly reducing the number of model parameters and inference complexity while maintaining high performance. Gou et al. provided a comprehensive review of the development of knowledge distillation, pointing out that it is not only applicable to classification tasks but also demonstrates broad adaptability and scalability in tasks such as object detection, segmentation, and semantic reasoning [56].

Based on the source and type of distilled information, mainstream methods can be categorized into two types: logit distillation and feature distillation.

Logit distillation guides the training of the student model by learning the soft labels (typically the probability distribution of the softmax layer) output by the teacher network. This approach was first proposed by Hinton, and the advantage of logit distillation lies in its ability to provide more granular category relationship information, effectively alleviating overfitting issues. In recent years, this method has also been applied to more complex task scenarios, such as decision planning and code representation modeling, in semantic reasoning tasks [57].

Feature distillation focuses on the transfer of intermediate layer representation information. This method minimizes the difference between the feature maps outputted by the teacher and student networks at specific layers (such as the backbone, FPN, and head), enabling the student model to acquire stronger semantic expression capabilities. Heo et al. systematically restructured and classified the feature distillation, proposing a distillation framework centered on activation re-organization and adaptive scaling, enhancing the distillation efficiency [58]. Shu et al. introduced a channel-wise distillation mechanism, improving performance in dense prediction tasks such as object detection and segmentation by applying weighted distillation to important feature channels [59]. Liu et al. further enhanced the generalization capability of feature transfer by preserving diversity between channels [60].

Additionally, Yue et al. proposed the Matching Guided Distillation strategy, which utilizes semantic alignment between teacher and student models for distillation, significantly improving the detection performance while maintaining semantic structural consistency [61].

In object-detection tasks, KD techniques are widely used to improve the detection accuracy of lightweight models (such as YOLO-tiny, MobileNet-SSD, etc.), especially in resource-constrained environments (such as edge computing and embedded systems). For example, the Mimic model proposed by Li et al. effectively improves the accuracy and stability of single-stage and multi-stage detectors by applying distillation losses at key locations such as RPN and RoIHead [62]. Tung and Mori proposed a distillation strategy that preserves the similarity structure between samples, further enhancing the category distribution consistency and discrimination ability [63]. Additionally, in transformer-based detectors, knowledge distillation is used to guide the alignment of attention distributions and bounding box matching strategies, serving as a key method to accelerate convergence and improve small-object-detection performance.

## 3. Method

The design objective of MRC-DETR is to significantly reduce the computational overhead while maintaining model detection accuracy, thereby enabling the efficient real-time detection of PEPPE. The model aims to enhance feature extraction and fusion capabilities, particularly addressing the challenges of detecting small and occluded objects in complex environments, such as high-altitude operations and power maintenance. To achieve this goal, MRC-DETR balances accuracy and lightweight design in its structural design, driven by the following two key design motivations:

1. MRC-DETR achieves the efficient aggregation and enhancement of features at different scales by constructing a multi-level backbone network based on the Moga-CSP module and a feature fusion structure (MRC-Fusion Encoder) incorporating the Multiscale Enhanced Boundary Attention (MEBA) module. The MEBA module enhances the model’s perception of small and edge objects in complex backgrounds through a multi-scale boundary enhancement strategy, significantly improving the feature representation quality.

2. Given the resource constraints on deployed devices in power operation environments, MRC-DETR introduces a distillation mechanism to transfer knowledge from a more complex and accurate teacher model to a lightweight student model. The distillation process employs a dual strategy combining feature-based knowledge distillation and logit-based distillation, enabling the student model to maintain detection performance close to the teacher model while offering advantages in inference speed and resource consumption.

### 3.1. Model Structure Design

**Figure 3:**
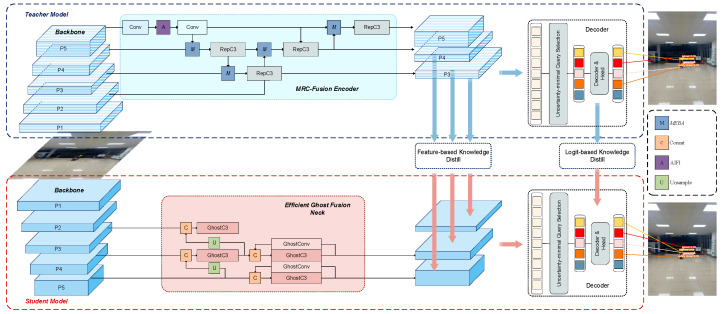
Structure of the MRC-DETR model.

The overall structure of MRC-DETR is shown in Figure 3. MRC-DETR adopts a teacher–student architecture to improve the PEPPE detection performance in complex power operation scenarios. The teacher model constructs the MRC-Fusion Encoder, which combines a multi-scale feature enhancement module (MEBA), an AIFI attention mechanism, and a deep convolutional module (RepC3) to strengthen the expression capabilities of small objects and boundary information. The multi-scale features outputted are not only used for final decoding but also transferred to the student model through feature-level and logit-level knowledge distillation, guiding it to learn more precise feature representations.

The student model adopts a lightweight design, embedding the Efficient Ghost Fusion Neck, which uses the Ghost module for efficient feature reconstruction and fuses multi-scale features to adapt to resource-constrained scenarios. Although MRC-DETR adopts a distillation network framework, resulting in a seemingly large and complex model, only the small “student” network is deployed on the terminal device. This approach ensures the efficiency and accuracy of the final model.

#### 3.1.1. Backbone

Power Engineering Personal Protective Equipment detection requires high accuracy in identifying details and boundaries, high accuracy in identifying equipment locations, and strong adaptability to complex backgrounds. The model must balance local details and the overall structure under different lighting conditions and angles to ensure the accuracy of target identification while maintaining robustness in complex visual environments.

**Figure 4:**
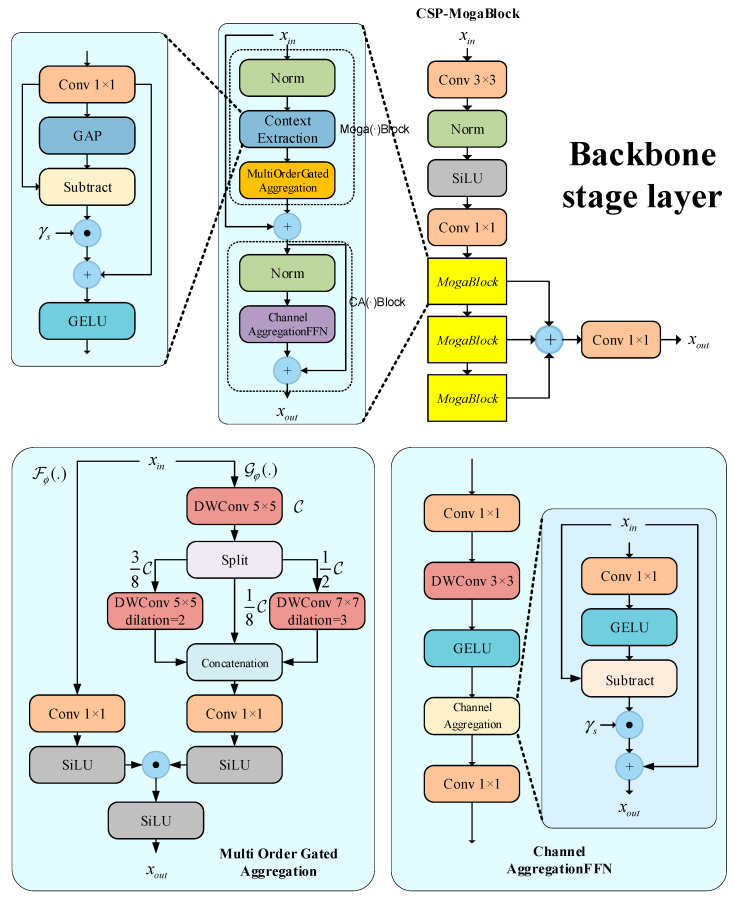
Backbone network structure diagram.

The proposed backbone network effectively captures multi-scale, multi-order features through its hierarchical structure. As shown in Figure 4, the backbone consists of multiple Layer Stages, each containing a convolutional module (3 × 3 convolution, BatchNorm, and SiLU activation function) and a CSP-MogaBlock. Each CSP-MogaBlock comprises three Mogablocks, enabling the network to progressively extract and contextualize features at different resolutions and scales.

The structure of Mogablocks is divided into gated aggregation units and channel aggregation units. In the gated aggregation units, the feature decomposition module (FD) is used to extract local and global features:

(1) Z=X+Moga (FD(Norm(X)))

Among them, *FD*(*·*) is a feature decomposition module, and *Moga*(*·*) is a multi-order gate aggregation module, including gate *Fφ*(*·*) and context branch *Gψ*(*·*). In order to force the network to focus on multi-order interactions, *FD*(*·*) can be used to dynamically exclude trivial interactions.

(2) $$Y={\mathrm{Conv}}_{1\times 1}(X)$$

(3) $$Z=\mathrm{GELU}\left(Y+\gamma_{s}⊙\left(Y-\mathrm{GAP}(Y)\right)\right)$$

In this context, *γ_s_* is the scaling factor, and GAP(·) denotes global average pooling. This module enhances feature diversity through reweighted complementary interactions. To capture features at different scales, three parallel deep convolutions with different expansion rates are used for high-, medium-, and low-level features, respectively. The output features of these convolutions are concatenated into a multi-scale feature map *Y_C_*. The gated aggregation mechanism uses the SiLU activation function to adaptively fuse features, and the final output is as follows:

(4) $$Z=\mathrm{SiLU}(\mathrm{Conv}1\times 1(X))⊙\mathrm{SiLU}(\mathrm{Conv}1\times 1(Y_{C}))$$

The channel aggregation module (CA) is used to enhance feature interaction between channels. It includes channel normalization, multi-scale depth convolution, and channel mixing mechanisms:

(5) $$Y=\mathrm{GELU}\left(\mathrm{DW}3\times 3\left(\mathrm{Conv}1\times 1\left(\mathrm{Norm}(X)\right)\right)\right)$$

(6) $$Z={\mathrm{Conv}}_{1\times 1}(\mathrm{CA}(Y))+X$$

CA(·) is defined as follows:

(7) $$\mathrm{CA}(X)=X+\gamma_{c}⊙\left(X-\mathrm{GELU}(XW_{r})\right)$$

where *γ_c_* is the channel scaling factor. Each stage of the backbone consists of multiple Moga Blocks and a channel aggregation block. This structure enables the network to extract features at different scales in a hierarchical manner. The output of each stage is downsampled and fed into the next stage for further processing.

On this basis, CSP-MogaBlock, as an extension to MogaBlock, refers to the design idea of a CSP module and contains multi-layer stacked MogaBlock modules in its structure, to realize deeper feature transfer and more effective parameter sharing. In the forward propagation process, CSP-MogaBlock contains three Moga-Block modules; then, the final output is x = C2f(x, M_1_, M_2_, M_3_), where M_i_ is the i-th MogaBlock.

#### 3.1.2. Fusion Encoder

The MRC-Fusion Encoder is a key component of MRC-DETR, designed to enhance the model’s ability to perceive small and occluded objects in complex scenes through multi-scale feature fusion. This module stacks multiple RepC3 units for deep feature extraction while introducing skip connections between different scales to enhance the transmission and fusion of semantic information. To further improve the recognition accuracy in boundary regions, we designed a Multiscale Enhanced Boundary Attention (MEBA) module, which performs the fine-grained modeling of low-level (L), medium-level (M), and high-level (H) features. Through an attention mechanism, it dynamically adjusts the fusion weights of these features, thereby strengthening the feature expression capabilities in boundary-sensitive regions. This fusion strategy not only improves the overall detection performance but also provides a more distinctive feature foundation for subsequent knowledge distillation.

**Figure 5:**
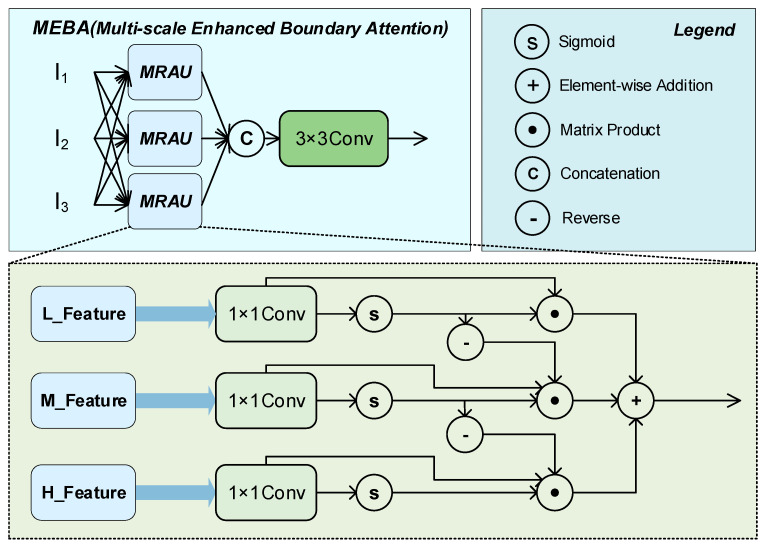
MEBA module structure diagram.

The MEBA Module Structure Diagram is shown in Figure 5. The MEBA includes several multiple recalibration attention unit (MRAU) blocks that adaptively extract mutual representations from three inputs (I1, I2, and I3) before fusion. As shown in Figure 1, shallow, mid-level, and deep-level information are inputted into the three MRAU blocks in different ways to compensate for the missing spatial boundary information of high-level semantic features and the missing semantic information of low-level features. Finally, the outputs of the three MRAU blocks are concatenated together after 3 × 3 convolution. This aggregation strategy achieves a robust combination of multiple features and refines the rough features.

The functional process for MRAU blocks can be represented as follows:

(8) $$T_{1}^{\prime }=W_{\alpha }(T_{1}),T_{2}^{\prime }=W_{\beta }(T_{2}),T_{3}^{\prime }=W_{\gamma }(T_{2})$$

(9) $$U(T_{1},T_{2},T_{3})=T_{1}+T_{1}^{\prime }⊙T_{1}+T_{2}^{\prime }⊙T_{2}⊙(⊖(T_{1}^{\prime }))+T_{3}^{\prime }⊙T_{3}⊙(⊖(T_{1}^{\prime }))$$

In this module, T1, T2, and T3 represent three input features at different scales. We employ three learnable linear mapping functions, *W_α_*, *W_β_*, and *W_γ_* (implemented as 1 × 1 convolutions), to compress the channel dimensions of the input features to 32, thereby generating corresponding attention feature maps. The Sigmoid function is used to produce attention weight maps, while the ⊙ symbol denotes element-wise multiplication.

The notation ㊀(*T1′*) represents the inversion of the attention map, which serves to emphasize background regions or suppress specific areas. Through the differential operation on feature T1, more refined and complete prediction results can be extracted from the coarse initial estimation.

In this process, the 1 × 1 convolution is treated as a linear mapping operation. All parameters, *W_α_*, *W_β_*, and *W_γ_*, are trainable parameters within the network and are automatically optimized during model training, thus eliminating the need for the manual setting of weight coefficients α, β, and γ. Thus, the MEBA output can be represented as Z = C_3×3_(*Concat*((k_1_, k_2_, k_3_)), where k_i_ = *MPAU_i_*(F_1_, F_2_, F_3_), and C_3×3_ is the 3 × 3 convolution with batch normalization and the ReLU activation layer. F_1_, F_2_, and F_3_ are three different dimensional features in the backbone. This multi-scale feature aggregation and recalibration mechanism enables MEBA to accurately identify and localize the safe wearing of power equipment, which significantly improves the detection performance.

### 3.2. Loss Function

In the real world, the working background of electric workers exists in the field environment, and this complex environment often leads to the degradation of the bounding box localization ability for target detection. Therefore, we introduce Focaler-WIoU as the loss function of MRC-DETR, aiming to improve the model’s ability to focus on difficult samples, and at the same time, not to let the model “count” too much in the low-quality bounding box regression.

The WIoU (Wise IoU) loss function aims to improve object-detection performance by adjusting the gradient gain of the anchor frame, especially in complex environments such as grid operations, dealing with occlusions, and small objects in images. In WIoU, the quality of the anchor frame is described using outliers, where smaller outliers indicate higher quality. The authors assign smaller gradient gains to anchor frames with lower outliers to focus the bounding box regression on anchor frames of medium quality. Smaller gradient gains are assigned to anchor frames with larger outliers, thus effectively preventing low-quality samples from generating significant detrimental gradients. The formula is calculated as follows:

(10) $$\beta =\frac{L_{IoU}^{*}}{L_{IoU}}\in [0,+\infty )$$

(11) $$L_{WIoU}=rR_{WIoU}L_{IoU},r=\frac{\beta }{\delta \alpha^{\beta -\delta }}$$

(12) $$R_{WIoW}=\exp(\frac{{\left(x-x_{gt}\right)}^{2}+{\left(y-y_{gt}\right)}^{2}}{{\left(W_{g}^{2}+H_{g}^{2}\right)}^{*}})$$

Among them, *β* is defined as the outlier degree to describe the quality of the anchor box, and δ and α are both adjustable parameters used to control the outlier degree. *R_WIoU_* ∈ [1, e) significantly amplifies the L_IoU_ for anchor boxes of average quality. *LI_oU_* ∈ [0, 1] significantly reduces *R_WIoU_* for high-quality anchor boxes. It also reduces attention to the center-point distance when anchor boxes overlap well with object boxes.

However, WIoU is deficient in dealing with the distribution of hard and easy samples and cannot pay sufficient attention to hard-to-detect samples. To address this problem, we introduce Focaler-WIoU inspired by Focaler-IoU. Focaler-IoU effectively improves the performance of the model in different detection tasks by paying more attention to hard samples. Focaler-WIoU, on this basis, dynamically adjusts the gradient gain of the samples to prioritize hard samples while taking care of the easy samples, thus realizing a more balanced and effective training process. This improvement enables Focaler-WIoU to significantly improve the detection accuracy and robustness in complex environments, especially in scenes with many occlusions and small targets.

The formula of Focaler-WIoU is as follows:

(13) $$L_{Focaler-WIoU}=L_{WIoU}+IoU-IoU^{Focaler}$$

(14) $$IoU^{Focaler}=\left\{\begin{array}{l} 0,IoU<d\\ \frac{IoU-d}{u-d},d<<IoU<<u\\ 1,IoU>u \end{array}\right.$$

where *IoU* is the original IoU value and [*d*, *u*] ∈ [0, 1]. By adjusting the values of *d* and *u*, we can make IoU-focaler focus on different regression samples.

### 3.3. Knowledge Distillation

In the real world, devices such as surveillance cameras, drones, etc., are often used to monitor the wearing of PPE by power personnel in real time; however, specialized computing devices such as graphical processing units, tensor processing units, and high-performance computing clusters are rarely available, so deploying an object-detection model is challenging. Inspired by knowledge distillation techniques, we use a hybrid distillation approach to reduce network parameters to facilitate deployment on resource-constrained devices with a two-part strategy: (1) logic distillation and (2) feature distillation.

#### 3.3.1. Logic Distillation

Logic distillation focuses on aligning the student model with the teacher model’s output predictions. This approach is particularly well suited for target detection tasks because it allows the student model to mimic the teacher model’s predictions in terms of classification and localization.

Logic distillation loss consists of the following three components:

Categorical Loss *Lcls*: a binary cross-entropy loss was used to measure the similarity of categorical predictions between the student and teacher models, treating the categorical predictions of the teacher model as soft labels and encouraging the class distributions of the student model to be close to them.

(15) $$L_{cls}=\frac{1}{N}\sum_{i=1}^{N}BCE(\sigma (S_{score}),\sigma (t_{score}))$$

where *σ* denotes the Sigmoid function and sscore and tscore are the predicted scores of the student and teacher models, respectively.

Boundary box L1 loss *L_L1_*: The L1 loss was used to measure the difference between the student model and the teacher model bounding box predictions. This loss guides the student model to more closely approximate the bounding box output of the teacher model.

(16) $$L_{L1}=\frac{1}{N}\sum_{i=1}^{N}\left||s_{bbox}-t_{bbox}|\right|$$

IoU loss *L_IoU_*: To capture the IoU differences in bounding box predictions between student and teacher models, generalized IoU (GIoU) loss is used. This helps the student model to align with the bounding box of the teacher model in terms of location and shape.

(17) $$LIoU=\frac{1}{N}\sum_{i=1}^{N}\left(1-GIoU(s_{bbox},t_{bbox})\right)$$

Combining the above three losses and setting the weighting factors *α*, *β*, and *γ*, respectively, the final logical distillation losses are as follows:

(18) $$L_{logic}=\alpha L_{cls}+\beta L_{L1}+\gamma L_{IoU}$$

With this logic distillation strategy, the student model better simulates the predictions of the teacher model based on both the classification and edge localization tasks, resulting in improved detection accuracy.

#### 3.3.2. Feature Distillation

In addition to logic distillation, feature distillation allows student models to learn from the intermediate feature representations of teacher models. In this study, we utilize Channel-Wise Distillation (CWD) [49], which aligns the feature responses of the teacher and student networks at the channel level, thereby effectively transferring the spatial attention and semantic structure.

We specifically apply Class-Wise Distillation (CWD) to three detection head-connected layers in both the teacher and student models—layers that directly contribute to the generation of bounding box predictions. These layers were selected because they represent critical stages in the model pipeline, where rich semantic features and localization information are integrated to produce the final object-detection outputs.

We specifically select layers 15, 17, and 19 from the teacher model, as well as layers 15, 18, and 21 (as shown in Figure 3) from the student model, for feature distillation. These layers are directly connected to the detection heads and represent various spatial scales and semantic levels within the feature hierarchy. They correspond to the final stages of feature extraction that are responsible for detecting small, medium, and large objects. The slight differences in layer indices reflect the structural simplification of the student model; however, the selected layers are functionally aligned. By choosing these layers, we ensure that the student model receives multi-scale and semantically rich supervision from the teacher, thereby preserving the critical knowledge necessary for object classification and localization. This targeted distillation strategy enhances the student model’s ability to generalize across different object sizes and improves the overall detection accuracy.

To enable a meaningful comparison and alignment at the channel level, we apply softmax normalization across the spatial dimensions of each feature channel in the teacher model. This normalization treats each channel as a spatial probability distribution, emphasizing discriminative regions and enhancing the comparability of features between the teacher and student representations.

By aligning key detection-related layers using Class-Wise Distillation (CWD), we ensure that the student model inherits the teacher’s high-level spatial awareness and channel-wise semantic discrimination. This inheritance is crucial for tasks such as PEPPE detection, which involves small and overlapping objects in complex scenes.

Using the Kullback–Leibler (KL) scatter, the CWD loss measures the difference between teacher and student features over channels. This loss encourages students to capture similar spatial patterns and semantic information by minimizing the information difference between teacher and student feature representations.

(19) $$L_{cwd}=\sum_{c=1}^{C}\sum_{i=1}^{H\times W}KL(softmax(y_{t})||Logsoftmax(y_{s}))$$

where *KL* denotes KL scatter, and *Softmax* and *LogSoftmax* normalize the feature map in the channel dimension, respectively.

The total feature distillation loss is the sum of all selection layer CWD losses. The total loss of the student model is as follows:

(20) $$L_{total}\;=L_{logic}\;+\lambda L_{CWD}$$

where *λ* is a weighting factor for the equilibrium logistic distillation and characteristic distillation contributions.

## 4. Experiments

### 4.1. Dataset

**Figure 6:**
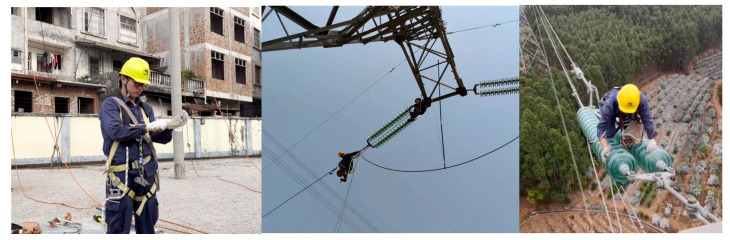
Examples of PEPPE datasets.

**Figure 7:**
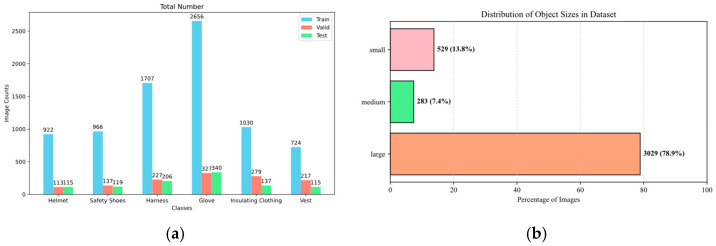
(**a**) Schematic representation of each classification and the number of classifications in the PEPPE dataset. (**b**) Statistical chart of the sizes of annotation boxes in the dataset.

We have introduced the Power Engineering Personal Protective Equipment (PEPPE) dataset, a specially curated collection of 3841 images documenting the actual working conditions of power workers in real-world scenarios, such as substations and high-altitude work sites. An example of the dataset is shown in Figure 6. The dataset includes six key categories of personal protective equipment—safety helmets, insulated gloves, safety belts, etc.—with images featuring complex backgrounds, varying degrees of occlusion, and lighting conditions to reflect the challenges faced in the real-world power industry. The dataset is divided into a training set (3073 images), a validation set (384 images), and a test set (384 images) in an 8:1:1 ratio (the number and proportion of each category are detailed in Figure 7a, and the number of the sizes of annotation boxes in the dataset are detailed in Figure 7b), ensuring the balance of each subset. The dataset is available via https://github.com/happyWEEKday/PEPPE-data.git (accessed on 30 June 2025).

### 4.2. Evaluate Metrics and Training

#### 4.2.1. Experimental Parameter Settings

The Windows 11 operating system was used for this experiment. The CPU configuration was 16 vCPU Intel(R) Xeon(R) Platinum 8352V CPU @ 2.10 GHz (Intel, Santa Clara, CA, USA), and a 24 GB NVIDIA GeForce RTX 4090 graphics card GHz (Nvidia, Santa Clara, CA, USA) was used for the GPU. The training virtual environment was developed using Anaconda3, and the code was run on Python 3.8, PyTorch 2.0.1, and Torchvision 0.15.2. Some of the hyperparameters used in MRC-DETR training are shown in Table 1.

To enhance the robustness and generalization of the model, a series of online data-augmentation techniques was applied during training, encompassing color, geometric, and compositional transformations. The specific augmentation parameters are summarized in Table 2. These augmentation strategies were intentionally selected to replicate the real-world variability in power operation scenarios while maintaining the spatial and structural integrity of PEPPE components—an essential consideration in safety-critical applications.

To evaluate model performance, we employ standard detection metrics including the Precision, Recall, AP50 (average precision at 50% IoU threshold), and AP50:95 (averaged across IoU thresholds from 50% to 95%). For the computational analysis, we measure Parameters (total trainable weights) and FLOPS (floating-point operations) to assess the model complexity and efficiency. Real-time capability is quantified through FPS (frames per second), tested on an NVIDIA RTX 4090 with a 640 × 640 input resolution after proper warm-up and synchronization. All timing metrics were averaged over 1000 inference runs with a batch size of 1, while computational metrics were calculated using the thop library.

#### 4.2.2. Result Visualization

**Figure 8:**
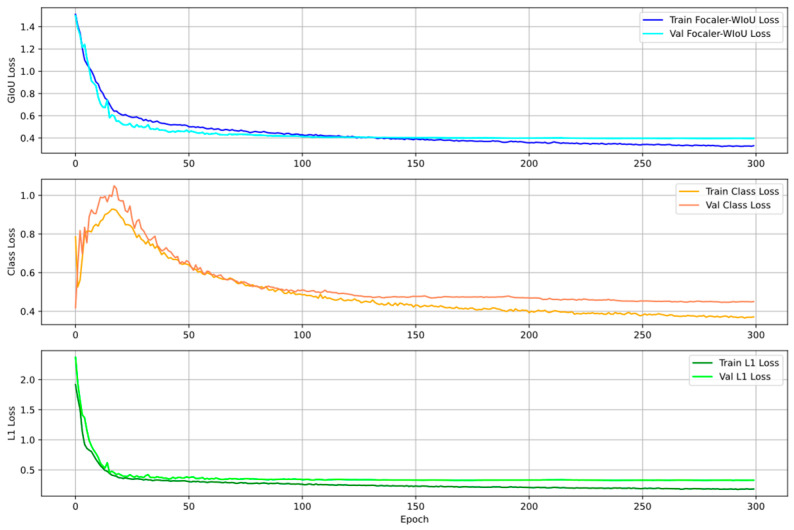
Loss function variation during the MRC-DETR training process.

Figure 8 illustrates the training and validation losses of the model, which include the Focaler-WIoU loss, classification loss, and L1 loss. As shown, all loss components demonstrate a distinct downward trend and stabilize after approximately 80 epochs, indicating effective training without any signs of overfitting.

**Figure 9:**
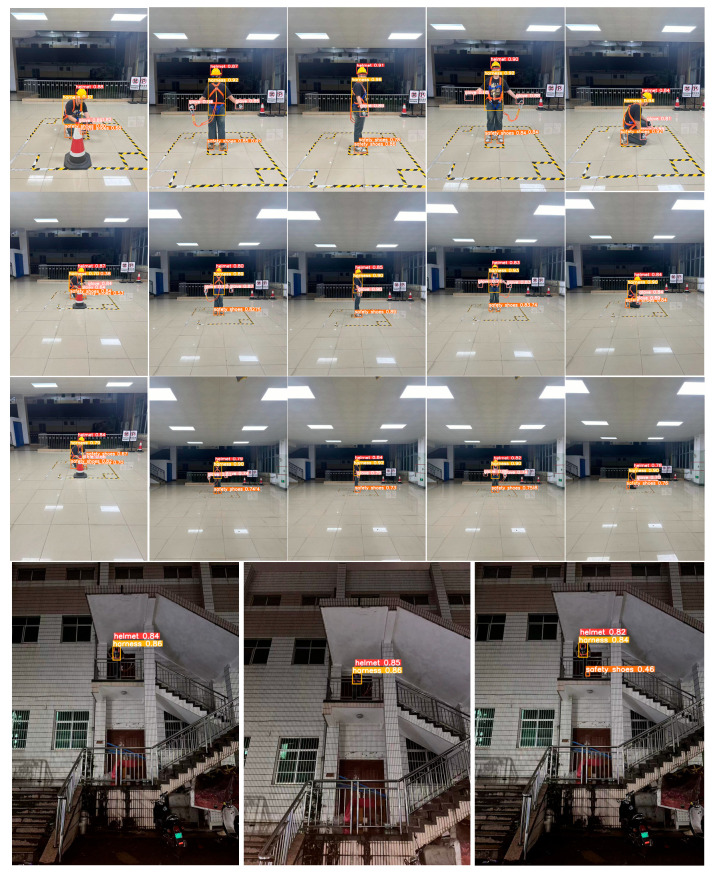
Visualization of the test set detection results.

To comprehensively validate the model’s performance under realistic conditions, we constructed a dedicated test dataset capturing volunteers performing typical work behaviors while wearing PEPPE. The evaluation protocol incorporated multiple challenging dimensions: (1) varying detection distances (3 m, 5 m, and 7 m) to assess scale robustness, with results showing sustained high precision (>94%) and recall (>93%) at 3 m–5 m distances, and only a moderate performance decline at 7 m due to occlusion and resolution limits; (2) diverse worker postures (standing/crouching) and viewpoints (front/side/back); and (3) controlled environmental variations including indoor/outdoor lighting conditions (1440 × 1080 resolution). As demonstrated in Figure 9 and Table 3, this rigorous multi-factor testing framework confirms the model’s stable detection capability across spatial, postural, and illumination challenges characteristic of real-world deployment scenarios.

**Figure 10:**
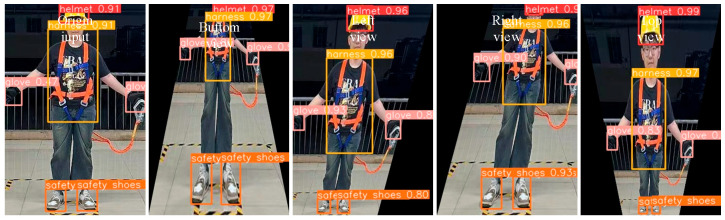
Multi-view visualization of detection results and detection effect diagram of the test set.

To assess the model’s adaptability to complex spatial configurations, we conducted additional experiments utilizing synthetic viewpoint transformations. Based on the original front- and side-view images, we employed perspective transformation techniques using OpenCV to generate virtual views from the left, right, top-down, and bottom-up perspectives. These transformations simulate real-world camera placements, such as those encountered in mobile patrol or aerial inspection scenarios. The transformed images were inputted into the model, and the visualized detection results (as shown in Figure 10) confirmed that the performance remained stable across various perspectives, demonstrating the model’s capability for spatial generalization.

Furthermore, we analyzed the detection performance across various PEPPE categories, as summarized in Table 4. Categories such as insulating clothing and vests achieved the highest mean Average Precision at IoU thresholds of 0.50 to 0.95 (mAP50:95) scores of 0.793 and 0.806, respectively. This superior performance is likely attributed to their high-contrast textures, consistent shapes, and relatively large visual footprint. In contrast, categories such as harnesses (mAP50:95 reaches 0.654) and gloves (mAP50:95 reaches 0.602) demonstrated moderate performance, which was influenced by frequent occlusions, irregular shapes, and color similarities with background elements. Safety shoes exhibited the lowest performance, with an mAP50:95 score of 0.491, primarily due to their small size, frequent partial occlusions, and limited visual distinctiveness when viewed from typical surveillance angles.

### 4.3. Ablation Experiments

#### 4.3.1. The Contribution of Different Modules

We conducted ablation experiments to demonstrate that the inclusion of MRC-Fusion Encoder, Moga-CSPNet, and Focaler-WIoU improves the performance of the model based on the PEPPE dataset. The results of the ablation experiments (shown in the Table 5) indicate that each module produces a stable and significant synergistic effect on the model performance: the introduction of the MRC-Encoder, Moga-CSPNet backbone, and Focaler-WIoU loss functions alone brings 0.5%, 1.6%, and 0.6% of AP50 enhancement, respectively, whereas the combined effect of all three achieves a 95.9% AP50, a 3.4% improvement from the baseline, surpassing the linear superposition of the independent improvements in each module (2.7%), confirming the synergistic enhancement between the multiscale feature encoder and the optimized backbone network and the improved loss function.

**Figure 11:**
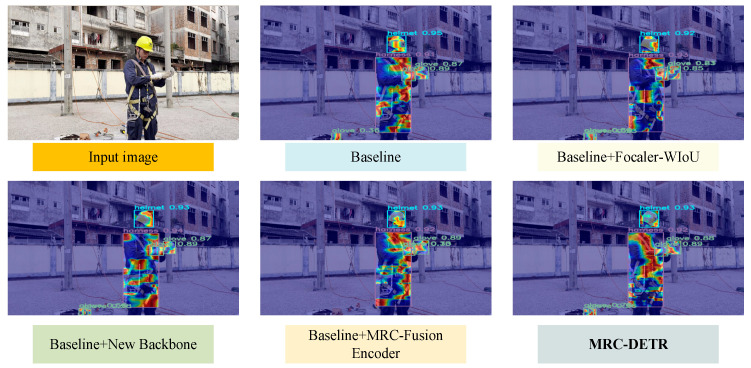
Visualization of ablation experiments.

Figure 11 shows the feature maps outputted by each module. By progressively integrating the Focaler-WIoU, Moga-CSPNet, and MRC-Fusion Encoder modules into the baseline model, the object-detection performance has been significantly optimized. First, the Focaler-WIoU module enhances the boundary accuracy, allowing detection boxes to more precisely fit the target contours and concentrate more on critical areas such as the head and arms. Second, with the introduction of the Moga-CSPNet, the model’s feature-extraction capability is markedly improved, capturing target features comprehensively and maintaining high resolution even against complex backgrounds. Then, the MRC-Fusion Encoder module further enhances feature fusion, enabling balanced attention to target areas across different feature scales, resulting in clearer and more stable detection boxes, especially in challenging backgrounds. Ultimately, the MRC-DETR model, which integrates all these improvements, achieves the best performance, exhibiting the highest detection accuracy and a strong focus on target areas, showcasing the substantial performance boost brought by the synergy of all modules.

#### 4.3.2. Contributions of Different Loss Function Parameters

We conducted experiments on the contributions of different loss function parameters in Focaler-WIoU, and the results are shown in Table 6. The ablation studies on threshold parameter selection in the Focaler-WIoU loss function reveal critical insights into its optimization dynamics. As demonstrated in Table 6, the model attains peak performance when configuring the lower threshold near zero and upper threshold approaching 0.95, achieving outstanding AP50 and AP50:95 scores of 95.2 and 67.5, respectively. This optimal configuration enables the loss function to most effectively discriminate between positive and negative samples while maintaining robust suppression of low-quality predictions.

A performance analysis shows sensitive dependence on threshold parameter selection, particularly regarding the upper bound parameter. Reducing the upper threshold below optimal levels leads to measurable degradation in the AP50:95 performance, with experimental results indicating approximately 1.5% deterioration when the upper threshold decreases substantially. The underlying mechanism, as described in Equations (13) and (14), operates through the linear scaling of prediction weights based on bounding box IoU within the specified threshold range.

Crucially, the study demonstrates that overly conservative threshold settings—either through excessive elevation of the lower bound or excessive reduction of the upper bound—constrict the effective learning region. This contraction measurably impairs the loss function’s capacity to properly guide bounding box regression quality, as evidenced by the performance variations captured in our systematic parameter sweep. The observed behavior confirms the importance of maintaining appropriate threshold margins to balance sample discrimination and learning effectiveness.

#### 4.3.3. Contributions of Different Knowledge Distillation Methods

We evaluated multiple distillation methods using two configurations: feature-level and combined feature and logic distillation. Results (Table 7) show that Channel-Wise Distillation (CWD) with full supervision achieved optimal metrics, proving particularly effective for fine-grained PEPPE detection. The performance gains confirm that joint logic and feature distillation yields more robust student models.

### 4.4. Model Comparison

To thoroughly assess the effectiveness of the proposed MRC-DETR, we conducted comparative experiments with a variety of representative object detection models, including Faster R-CNN, SSD, several YOLOv8/10 variants, DETR-based models, and RT-DETR, as summarized in Table 8. No additional tuning was applied to the baselines beyond their originally reported configurations.

Among all the models tested, MRC-DETR achieves the highest AP50 score of 95.2, demonstrating its superior object-recognition capability based on the PEPPE dataset. In terms of efficiency, MRC-DETR operates at 321 FPS, which is significantly faster than most transformer-based models, including Deformable-DETR (44.4 FPS), DAB-DETR (53.5 FPS), and Conditional-DETR (56.6 FPS). Although RT-DETR, utilizing a lightweight ResNet-18 backbone, achieves the highest speed (391.9 FPS), its accuracy (AP50:95 reaches 0.625) is still lower than that of MRC-DETR. In addition, MRC-DETR maintains a compact model size of only 19.40 million parameters and 71.20 G FLOPs, which is among the lowest in the DETR-based models compared. For instance, DETR and Deformable-DETR require 41.56 M and 40.10 M parameters respectively, but yield lower AP50:95 scores (0.479 and 0.593). These results clearly demonstrate that MRC-DETR provides a well-balanced trade-off among accuracy, speed, and resource consumption, making it highly suitable for real-world deployment scenarios that require both reliability and efficiency, such as the safety inspection of power equipment.

We also conducted experiments on the COCO dataset, and according to the experimental results, our proposed MRC-DETR performs on the COCO benchmark dataset, although the results are not optimal, but it also shows that it has a stable generalization ability.

### 4.5. Comparisons with Other Methods

We compared MRC-DETR with existing PEPPE detection models, and the results are shown in Table 9. The methods, which were used for a comparison, had their model parameters set according to their provided parameters and were trained and validated using the PEPPE dataset. While individual methods may surpass MRC-DETR in precision or recall for specific categories, our model exhibits a more balanced trade-off across all targets. Notably, MRC-DETR outperforms prior approaches in critical equipment such as safety harnesses, helmets, and insulated gloves, with particularly high recall values—indicative of its superior detection sensitivity in complex scenarios. Moreover, in categories where other methods show higher recall (e.g., YOLOv7 for boots and helmets), MRC-DETR still maintains competitive precision and achieves a higher overall mean Average Precision (mAP), reflecting stronger generalization and robustness. This balance between precision and recall, coupled with its adaptability to dense scenes and diverse object scales, underlines MRC-DETR’s suitability for real-world PEPPE detection applications where both accuracy and coverage are critical.

## 5. Model Deployment

Traditional surveillance cameras face challenges in complex grid operation environments, particularly in remote or high-altitude locations, due to installation difficulties and limited signal transmission. To address this, we propose a mobile edge-computing solution using a TensorRT-accelerated neural network deployed on the Jetson Orin Nano (NVIDIA, Santa Clara, CA, USA)—a low-power, high-performance embedded platform.

We evaluated both the full-size MRC-DETR and its lightweight Ghost-DETR variant on the Jetson Orin Nano. Converting the PyTorch models to ONNX and optimizing for FP16 precision reduced the latency and memory usage. Despite initial challenges—such as MRC-DETR’s high computational cost and TensorRT’s limited support for custom deformable attention layers—we resolved these through knowledge distillation and module restructuring. The effect of the model deployment on Jetson Orin Nano is shown in Table 10. Notably, Ghost-DETR outperformed its teacher model (AP50:95 0.620 vs. 0.613), likely due to better edge-device adaptation.

**Figure 12:**
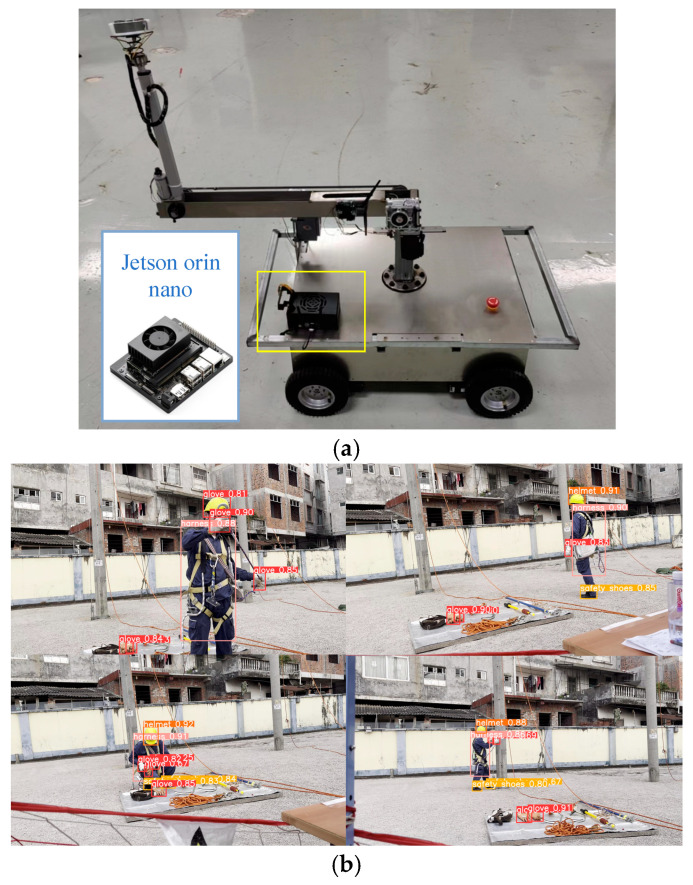
(**a**) Installation diagram. (**b**) Detection effect of model deployed on mobile devices.

Deployed in real-world scenarios, the system achieved 4.5 FPS with a 10 ms latency, enabling real-time PEPPE (Personal Electric Power Protective Equipment) compliance detection directly on-site (Figure 12). This eliminates reliance on central servers while improving safety and efficiency.

## 6. Summary

In this paper, we propose an MRC-DETR-based detection model for power operation and protection equipment, which effectively solves the problems of the insufficient detection accuracy, low efficiency, and limited applicability faced by the traditional methods in power operation scenarios through several technological innovations. First, we propose the MEBA module, which optimizes the multi-scale feature fusion mechanism and enhances the model’s ability to capture subtle features in complex environments. Second, we propose MRC-DETR, which significantly reduces the number of parameters and computational complexity of the model while maintaining the detection performance through the knowledge distillation technique, making it more suitable to be deployed on resource-constrained edge devices. Finally, we deploy MRC-DETR in edge devices to realize the real-time detection requirement, and the experimental results show that the model has important practical application value in power operation scenarios.

However, this research still has some limitations: current models can only detect the wearing status of protective equipment, but cannot effectively verify compliance with equipment wearing, such as critical safety hazards like helmets that are not securely fastened or seat belts that are not properly buckled. The accurate identification of these issues is critical to ensure safe power operations. In addition, the generalizability problem of MRC-DETR still needs to be improved. Future research should focus on developing methods for detecting equipment wear compliance and on improving the generalizability issue of the model to further improve the efficiency of PEPPE identification.

## Figures and Tables

**Table 1:** Training hyperparameter configuration.

Epoch	Batch Size	Workers	Optimizer	Learning Rate	Weight Decay	Momentum
300	16	4	AdamW	0.001	0.0005	0.937

**Table 2:** Data Augmentation Parameter.

Type	Parameter	Value	Description
Color	hsv_h	0.015	Hue adjustment range (fraction of 360°)
	hsv_s	0.7	Saturation adjustment scale (0 = grayscale, 1 = original)
	hsv_v	0.4	Value (brightness) adjustment scale (0 = black, 1 = original)
	translate	0.1	Image translation range (±fraction of image width/height)
	scale	0.5	Image scaling range (±gain)
	fliplr	0.5	Probability of horizontal flip (50% chance if 0.5)
Advanced	mosaic	0.01	Probability of mosaic augmentation (disabled if 0)
	mixup	0.01	Probability of mixup augmentation (disabled if 0)

**Table 3:** Detection results in diferent situations.

No.	Value	TP	FP	FN	Precision	Recall
1	3 m	93	5	5	0.951	0.948
2	5 m	91	5	7	0.945	0.932
3	7 m	89	6	8	0.932	0.920
4	occlusion	82	10	12	0.893	0.875

**Table 4:** Per-class detection performance metrics based on PEPPE items.

Classes	Precision	Recall	AP50	AP50:95
Safety shoes	0.933	0.895	89.8	49.1
Harness	0.966	0.948	96.0	65.4
Glove	0.931	0.913	92.2	60.2
Helmet	0.966	0.948	93.2	56.5
Vest	0.947	0.912	98.6	80.6
Insulating clothing	0.982	0.964	97.4	79.3

**Table 5:** Ablation experimental results.

Settings	MRC-DETR		L_Focaler-WIoU_	AP50
	MRC-Fusion Encoder	Moga-CSPNet		
Baseline (RT-DETR-r18)				92.5
MRC-Encoder	√			93.0
Moga-CSPNet		√		94.1
L_Focaler-WIoU_			√	93.1
All	√	√	√	95.2

Note: √ indicates adding this module on the basis of the Baseline.

**Table 6:** Loss function parameter tuning and performance comparison.

Parameters		AP50	AP50:95
d = 0	u = 0.95	95.2	67.5
d = 0.1	u = 0.9	93.7	61.5
d = 0.3	u = 0.7	93.7	60.4
d = 0.5	u = 0.5	92.8	60.5
d = 0.7	u = 0.3	93.8	61.3
d = 0.9	u = 0.95	93.3	61.7

**Table 7:** Comparative results of different knowledge distillation methods based on the MRC-DETR model.

Method		Evaluation			
Logic	Feature	P	R	map50	map50:95
		0.924	0.910	93.1	66
√		0.924	0.914	92.6	61
	chism [60]	0.930	0.904	92.3	60
	mgd [61]	0.931	0.916	93.1	62
	mimic [62]	0.934	0.924	93.4	66
	sp [63]	0.932	0.914	92.5	63
	cwd	0.941	0.925	93.6	61.0
√	chism	0.926	0.905	91.9	61
√	mgd	0.928	0.919	92.9	63
√	mimic	0.933	0.916	93.2	67
√	sp	0.931	0.913	92.5	68
√	cwd	0.934	0.934	94.0	62.0

Note: √ indicates adding this module on the basis of the Baseline.

**Table 8:** Object-detection model training results and comparison.

Method	Backbone	Params (M)	GFLOPs	FPS	PEPPE		COCO	
					AP50	AP50:95	AP50	AP50:95
YOLOv8l [32]	CSP	43.61	164.80	232.5	93.6	65.8	69.8	52.9
YOLOv8x	CSP	68.13	257.40	151.1	93.1	65.0	71.0	53.9
YOLOv10l	CSP	25.72	126.40	208.2	91.0	62.2	70.1	58.1
YOLOv10x	CSP	31.59	169.80	131.8	92.1	62.5	71.3	59.3
DETR-DC5	Resnet50	41.56	84.42	69.0	85.9	47.9	63.1	43.3
Deformable-DETR	Resnet50	40.10	170.00	44.4	91.6	59.3	65.2	46.2
DAB-DETR	Resnet50	43.703	89.77	53.5	93.6	63.7	66.0	46.9
Conditional-DETR	Resnet50	43.449	88.43	56.6	92.9	61.4	65.4	45.1
RT-DETR	Resnet50	41.96	129.65	165.3	92.5	62.2	71.3	53.1
RT-DETR	Resnet101	74.67	247.10	112.6	92.6	62.1	72.7	54.3
MRC-DETR	Moga-CSP	19.40 M	71.20	321.0	95.2	67.5	71.2	53.2

**Table 9:** The current PPE detection research results in precision and recall comparison.

Classes	Methods	P	R
Insulated shoes	MRC-DETR	0.933	0.895
	YOLOv4 + CioU (Xiang Y et al. [32])	0.913	-
	YOLOv7 ([64])	0.928	0.974
	Improved YOLOv5s with SIoU Loss Function ([65])	0.760	0.635
Harness	MRC-DETR	0.996	0.942
	Faster R-CNN + Harness detection network (Fang et al. [23])	0.800	0.980
	YOLOv3 + Openpose + Scene graph (Chen et al. [66])	0.934	0.829
	YOLOv5 + Openpose + 1D CNN (Jiaqi Li et al. [26])	0.997	-
Insulated glove	MRC-DETR	0.931	0.913
	Improved Deep Learning Algorithm (Chen et al. [67])	0.920	-
	YOLOv6x + SCAM + H-MHSA+ BiFPN (Wang et al. [24])	0.947	0.905
	YOLOv7 ([42])	0.943	0.874
	Improved YOLOv5s with SIoU Loss Function ([65])	0.726	0.690
Helmet	MRC-DETR	0.966	0.948
	Faster R-CNN (Fang et al. [68])	0.884	0.729
	YOLOv3 (Nath et al. [14])	0.793	-
	MobileNet with Multi-Scale Features and a Top-Down Module (Wang et al. [69])	0.894	-
	YOLOv5 + Openpose + 1D CNN (Jiaqi Li et al. [26])	0.953	-
	YOLOv3 + Openpose + Scene graph (Chen et al. [66])	0.973	0.918
	Improved Deep Learning Algorithm (Chen et al. [67])	0.960	-
	YOLOv7 ([42])	0.904	0.990
	Improved YOLOv5s with SIoU Loss Function ([65])	0.908	0.869
Insulating clothing	MRC-DETR	0.982	0.964
Vest	MRC-DETR	0.947	0.912
	YOLOv7 ([64])	0.748	0.962
	Improved YOLOv5s with SIoU Loss Function ([65])	0.914	0.803

- Not available.

**Table 10:** Model deployment parameters and deployment effects.

Device	Running Memory	Models	mAP50	AP50:95	FPS	Latency (ms)
Jetson Orin Nano	4 GB	MRC-DETR (teacher)	95.2	67.5	4.1	30
		Ghost-DETR (student)	94.0	62.0	4.5	10

## Data Availability

The PEPPE dataset is available via https://github.com/happyWEEKday/PEPPE-data.git (accessed on 30 June 2025).
